# Evaluation of early imaging response criteria in glioblastoma multiforme

**DOI:** 10.1186/1748-717X-6-121

**Published:** 2011-09-23

**Authors:** Adam Gladwish, Eng-Siew Koh, Jeremy Hoisak, Gina Lockwood, Barbara-Ann Millar, Warren Mason, Eugene Yu, Normand J Laperriere, Cynthia Ménard

**Affiliations:** 1 Faculty of Medicine, University of Toronto, Toronto, Canada; 2 Radiation Medicine Program, Princess Margaret Hospital, Toronto, Canada; 3 Department of Radiation Oncology, Liverpool Hospital, New South Wales, Australia; 4 University of New South Wales, NSW, Australia; 5 Department of Radiation Oncology, University of Toronto, Toronto, Canada; 6 Department of Medical Biophysics, University of Toronto, Toronto, Canada; 7 Department of Clinical Study Coordination and Biostatistics, Princess Margaret Hospital, Toronto, Canada; 8 Department of Medical Imaging, Princess Margaret Hospital, Toronto, Canada

**Keywords:** Glioblastoma Multiforme, Imaging response, radiotherapy, RECIST

## Abstract

**Background:**

Early and accurate prediction of response to cancer treatment through imaging criteria is particularly important in rapidly progressive malignancies such as Glioblastoma Multiforme (GBM). We sought to assess the predictive value of structural imaging response criteria one month after concurrent chemotherapy and radiotherapy (RT) in patients with GBM.

**Methods:**

Thirty patients were enrolled from 2005 to 2007 (median follow-up 22 months). Tumor volumes were delineated at the boundary of abnormal contrast enhancement on T1-weighted images prior to and 1 month after RT. Clinical Progression [CP] occurred when clinical and/or radiological events led to a change in chemotherapy management. Early Radiologic Progression [ERP] was defined as the qualitative interpretation of radiological progression one month post-RT. Patients with ERP were determined pseudoprogressors if clinically stable for ≥6 months. Receiver-operator characteristics were calculated for RECIST and MacDonald criteria, along with alternative thresholds against 1 year CP-free survival and 2 year overall survival (OS).

**Results:**

13 patients (52%) were found to have ERP, of whom 5 (38.5%) were pseudoprogressors. Patients with ERP had a lower median OS (11.2 mo) than those without (not reached) (p < 0.001). True progressors fared worse than pseudoprogressors (median survival 7.2 mo vs. 19.0 mo, p < 0.001). Volume thresholds performed slightly better compared to area and diameter thresholds in ROC analysis. Responses of > 25% in volume or > 15% in area were most predictive of OS.

**Conclusions:**

We show that while a subjective interpretation of early radiological progression from baseline is generally associated with poor outcome, true progressors cannot be distinguished from pseudoprogressors. In contrast, the magnitude of early imaging volumetric response may be a predictive and quantitative metric of favorable outcome.

## Background

In 1990, MacDonald et al [1] reported criteria for response assessment in glioma. Importantly, these criteria incorporated features such as time factors, degree of response of contrast-enhancing tumor using computed-tomography (CT)-based uni-dimensional World Health Organization (WHO) criteria [2], neurologic status and the use of corticosteroids. Although these criteria have become widely accepted, they have also been criticized for their limitations [3-5], including their inability to accurately assess complex tumor morphology, account for non-tumor factors that may cause contrast enhancement, reaction to local therapies [6], and lack of applicability to non-enhancing tumors. Furthermore, the phenomenon of 'pseudoprogression' observed in patients receiving concurrent chemo-radiotherapy [7-9], as well as the dilemma of 'pseudo-response' seen with some of the newer anti-angiogenic therapies [5,10], adds to the already complex challenge of early assessment as these phenomena can confound image interpretations.

The accurate and early prediction of response and/or progression remains important for several reasons. In principle, this may enable more objective evaluation and comparison of novel therapies [5]. Secondly, such a biomarker could be utilized as a surrogate endpoint in clinical trials, thus conferring the distinct advantage of earlier response prediction and greater opportunity to amend or institute alternate therapies, especially given the aggressive nature of Glioblastoma Multiforme (GBM). Thirdly, earlier imaging predictors could potentially allow the conduct of smaller clinical trials requiring fewer patients, enable earlier judgements about promising versus futile therapies, more expeditious regulatory approval for new drugs, and ultimately earlier application and translation of new therapies into clinical practice [11,12]. In reality however, the evidence for reliable imaging response thresholds that could ultimately influence therapeutic decision making is still lacking. Currently, response criteria are largely based on the response evaluation criteria in solid tumors (RECIST) guidelines [13,14], which were developed to standardize reporting of outcomes of clinical trials. Most recently, the Response Assessment in Neuro-Oncology (RANO) working group provided updated criteria for high-grade gliomas [15], but as of yet there is not analysis of these criteria as they relate to clinical endpoints such as overall survival and progression-free survival.

We embarked on a study investigating early structural and functional magnetic resonance imaging (MRI) evaluations of response in patients with GBM. As a first step, we sought to investigate the predictive value of standard structural imaging response criteria one month after the delivery of concurrent chemotherapy and radiotherapy (RT). We also undertook exploratory analysis of alternate structural imaging response thresholds that may better correlate with and/or predict for clinical outcomes.

## Methods

This study was approved by the institutional research ethics board. Patients were prospectively enrolled over a 26 month interval between May 2005 and July 2007. Patients were approached for enrollment if they met the following criteria: histological diagnosis of WHO grade IV Glioblastoma Multiforme; planned to receive definitive concurrent chemotherapy (temozolomide 75 mg/m^2 ^daily) and RT (60Gy in 30 fractions over 6 weeks) followed by adjuvant temozolomide chemotherapy (200 mg/m^2 ^× 5 days, monthly for 1 year or until progression); age ≥18 years; and ECOG performance status 0 or 1. Patients were excluded if they had contraindications to MRI, severe claustrophobia, or previous cranial radiotherapy. Relevant clinical and demographic information, including gender, age, diagnosis date, disease multi-focality, surgical status, and radiation treatment dates were also captured.

MRI acquisition was performed at the following time-points: Baseline (BL) post-operatively but prior to radiotherapy (RT); week 3 and week 6 of RT, 1 month after completion of RT, then every two months until evidence of clinical progression (defined below) or until 1 year of follow-up. All images were acquired using a 1.5 T GE Signa Excite scanner (GE Healthcare, Waukesha, WI, USA). The MRI acquisition protocol was performed as follows: Axial post-contrast axial T1-weighted fast-spin echo (FSE) (TE = 20 ms, TR = 416.66 ms, FA = 90°, BW = 122.109, slice thickness = 5 mm, slice spacing = 7 mm, 0.859 × 0.859 × 7 mm resolution).

Clinical and imaging end-points included: A) Time to Clinical Progression [CP] - interval between beginning of RT and CP defined as aggregate of clinical and radiological progression resulting in a change in patient management (for example, second-line chemotherapy, salvage surgery or palliative care); B) Overall Survival [OS] - defined as the interval between beginning of RT and death; C) Early Radiological Progression [ERP] - qualitative impression of any radiological progression from baseline to one month post-RT as defined by a radiation oncologist (CM), and D) Pseudoprogression - when ERP was present but the patient showed clinically stable disease for at least 6 months post-RT without a change in the adjuvant chemotherapy regimen.

Post-contrast axial T1-weighted FSE images were rigidly co-registered (mutual information algorithm) with the RT planning CT datasets using a commercial radiotherapy treatment planning system (Pinnacle^3 ^v7.6c and 8.1, Philips Radiation Oncology Systems, Madison, WI). A radiation oncologist (ESK, NL) delineated tumor volumes on the T1-weighted post-contrast MR images as defined by areas of abnormal contrast enhancement reflecting residual or recurrent tumor, whilst excluding areas of post-surgical change. All volumes were then reviewed and finalized by a diagnostic radiologist (EY).

Both longest diameter (axial, coronal, and sagittal planes) and 3D volumetric data (cc) were computed at baseline (BL) and one-month post RT. Progression was then assessed via RECIST criteria, a 20% increase in the longest tumor diameter or a 40% increase in volume (sums of diameters or volumes were used in the case of multi-focal disease). Disease response as determined by RECIST was defined as a 65% decrease in volume or a 30% decrease in diameter. The MacDonald criteria were also evaluated: progressive disease defined as a 25% increase in the largest tumor area (cm^2^) and responsive disease defined as a 30% decrease in largest area. Each patient was then classified in a binary fashion, as either having progressive or responsive disease based on these imaging thresholds. In addition, the following range of volume, area and diameter progression/response thresholds (see Additional File 1 - Table 1) were investigated including: Diameter - any increase; any increase or decrease up to > 5%, 15% or 30%; Area - any increase, any increase or decrease > 5%; 15% or 30%; and Volume - any increase, > 25% increase, any increase or decrease > 10%; 25%; or 50%.

Sensitivity and specificity values were calculated for each threshold using clinical progression-free survival at 1 year and overall survival at 2 years. Receiver-operator curves (ROC) were also constructed and statistical analysis was performed on the basis of work by DeLong et. al. [16]. Kaplan-Meier survival curves were created to analyze early progression, pseudoprogression and clinical progression as previously defined.

## Results

A total of 30 patients were prospectively recruited. One patient refused study procedures after enrollment and another 4 patients did not undergo MRI examination one month after RT, leaving a total of 25 patients from whom imaging data was analyzed. It should be noted that demographical and follow-up data was taken from all 29 patients followed, however only the demographics of the 25 patients analyzed in this study are reported here. The median age of patients enrolled was 56 years (15 men, 10 women, range 46 - 68 years). Five patients presented with multifocal disease. Tumor volumes at baseline ranged from 0.96 cm^3 ^to 143.2 cm^3^. The majority of patients were enrolled after gross total resection (n = 14), while 8 and 3 patients underwent partial resection and biopsy only, respectively.

The study cohort had a median follow-up of 26.3 months (range 13.3 - 37.7 months). Median survival was high at 26.7 months and median time to clinical progression was 7.5 months (range 1.5 mo. - 35.9 mo.).

**Figure 1:**
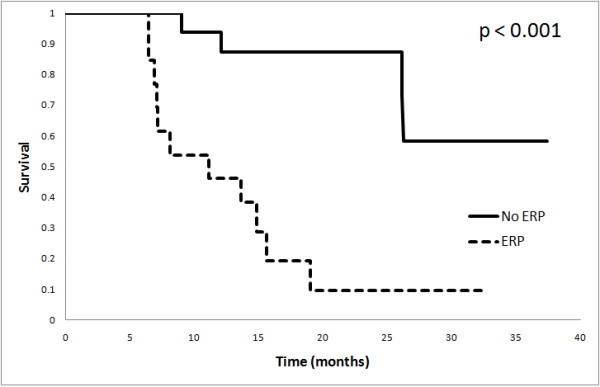
**Overall survival according to 1 month radiological progression status**: Overall. survival based on any early radiological progression (ERP), observed one month after RT.

**Figure 2:**
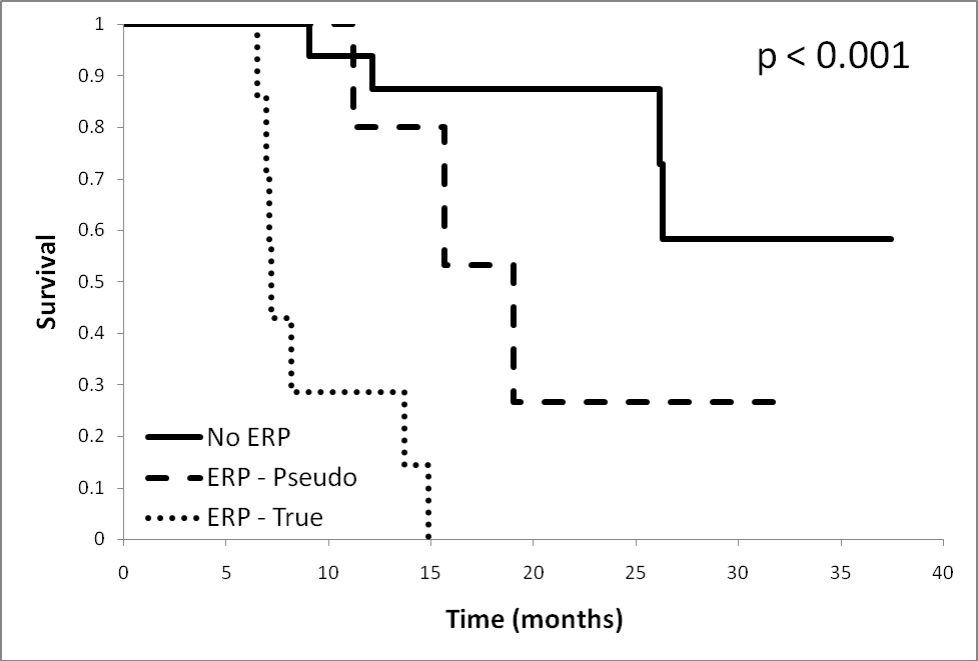
**Overall survival according to true vs. pseudo-progression status**: Overall survival. based on true *vs*. pseudo progression at one month.

A qualitative impression of any radiological progression (ERP) from baseline was found in 11 patients (40.0%), although only 2 patients strictly met the MacDonald criteria for progression at 1 month. Median survival for patients with ERP was significantly shorter than those without (11.2 mo vs. not reached, p < 0.001) (Figure 1). Of those with ERP, five were subsequently determined to have pseudoprogression (45.5% of ERP). Pseudoprogressors fared better than true early progressors, with a median survival of 19.0 months vs. 7.2 months (p < 0.001), (Figure 2)

**Figure 3:**
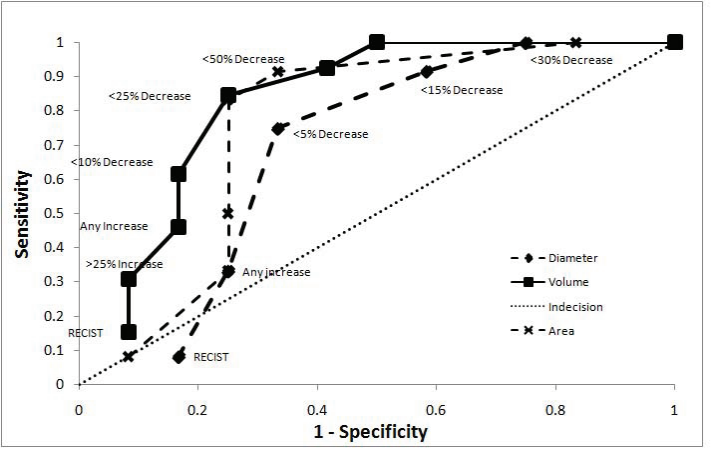
**Receiver-Operator Curve by Dimension Metric**: Receiver-operator curves for volume (solid, square), area (dashed, cross) and diameter (dashed, diamond) thresholds in predicting 2 year overall survival. Line of indecision is marked as a dotted line.

**Figure 4:**
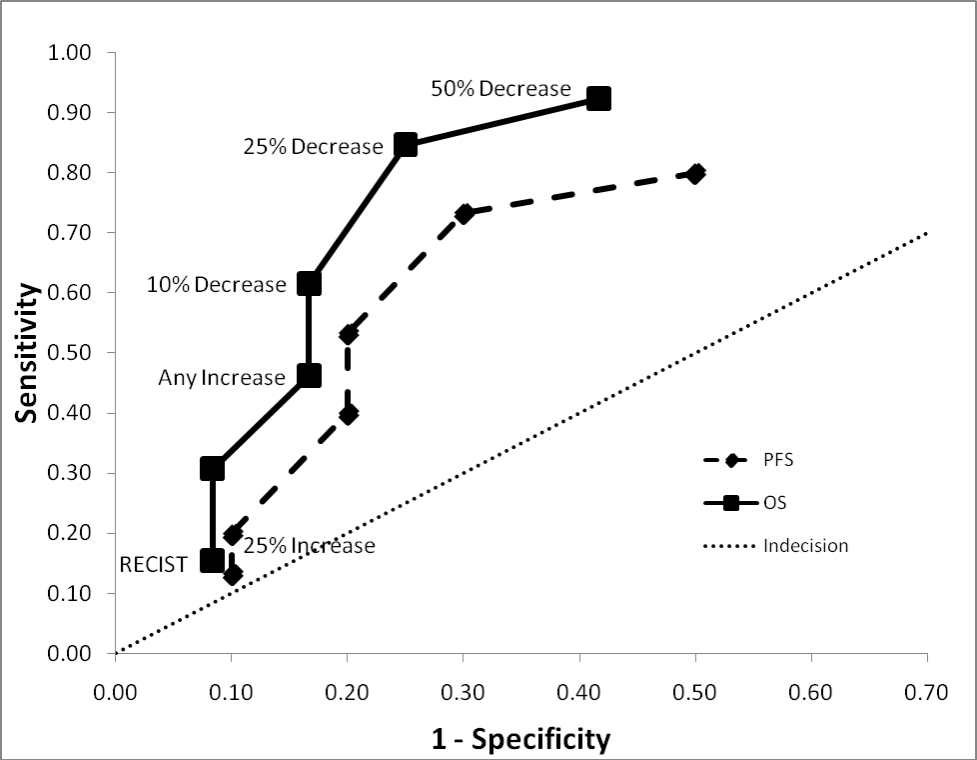
**Receiver-Operator Curve of Volume Metrics by Clinical End-point**: Receiver-operator curves for volume thresholds in predicting for 2 year overall survival (solid, square) and 1 year clinical progression-free survival (dashed, diamond). Line of indecision is marked as a dotted line.

**Figure 5:**
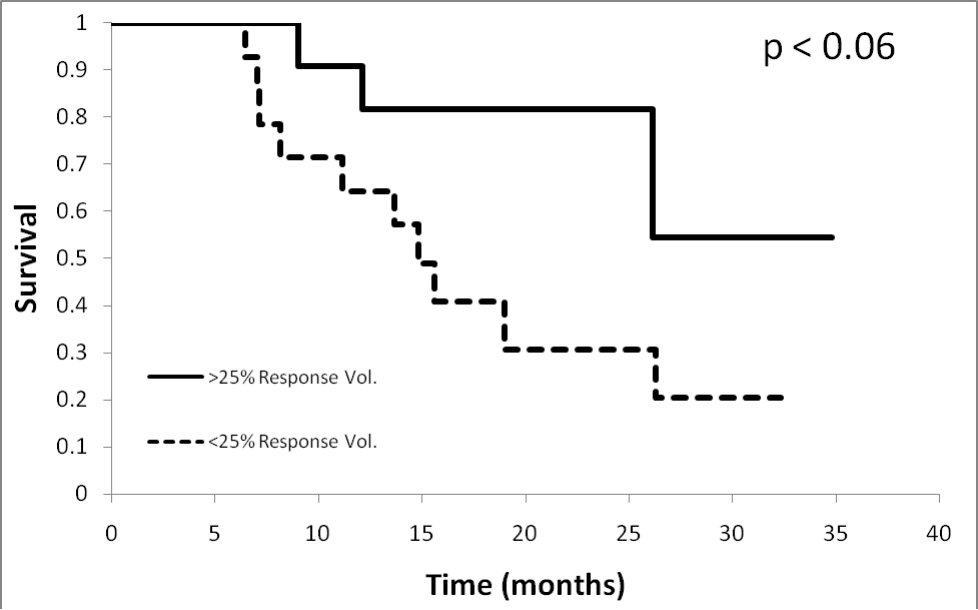
**Kaplan-Meier survival according to 25% Volume Response at 1 month**: Kaplan-Meier survival curve for patients with and without a > 25% response in tumour volume, one month after RT.

Sensitivity and specificity values were calculated for each response threshold, along with the positive and negative likelihood ratios (+LH; -LH) and the area-under-the-curve (AUC) for volume, area and diameter metrics (see Additional files 1, 2, 3 - Table 1, 2 and 3 respectively) in predicting for 2-year overall survival. The most sensitive tests were those measuring response, namely greater than 25% and 50% decreases in volume and 15% and 30% decreases in area and diameter. The most specific tests were those with the highest thresholds for progression, namely the RECIST criteria for both volume and diameter, and MacDonald criteria for area. In general, the volume measurements consistently performed better in every category than did the area and diameter metrics. This trend can also be visualized in Figure 3, receiver-operator curves plotting sensitivity vs. 1-specificity for the volume, area and diameter thresholds against overall survival at 2 years. The respective AUC's are 0.83 (0.59 - 0.94 95% CI), 0.76 (0.53 - 0.90 95% CI) and 0.69 (0.44 - 0.84 95% CI) for volume, area and diameter respectively. These values were significantly different from chance (AUC of 0.5) for both volume and area (p < 0.005 and p < 0.05, respectively) but not for diameter (p > 0.1). When comparing amongst AUC's there was no significant difference between volume, area or diameter, with the greatest trend seen between volume and diameter (p > 0.1). The two most prognostic thresholds were > 15% decrease in area (3.33 +LH, 0.22 -LH) and > 25% decrease in volume (3.38 +LH, 0.21 -LH). Figure 4 compares the receiver-operator characteristics of volume thresholds when predicting for progression-free survival at 1 year and overall survival at 2 years, demonstrating a trend that volume metrics to be more predictive of overall survival at 2 years than PFS at 1 year (AUC 0.83 vs. 0.70, p < 0.2). Figure 5 depicts Kaplan-Meier survival based on > 25% volume response at 1-month post RT nearing statistical significance (median survival 14.9 mo vs. not reached, p < 0.06).

## Discussion

The early and accurate prediction of response to cancer treatment through the application of imaging criteria has several potential advantages. Ideally, imaging thresholds would provide utility as surrogates for outcome over and above the more traditional measures including overall and progression free survival [17], allowing for more expeditious conduct of clinical trials (both phase II [18] and III). This in turn could lead to the earlier institution of alternate therapies that show a beneficial effect on outcome. This is particularly important in dealing with aggressive and rapidly growing malignancies such as GBM.

Our results show that across all thresholds, both progressive and responsive, volume was uniformly more predictive of OS and PFS as seen by the right shift of the diameter ROC curve in Figure 3 (AUC of 0.83 vs. 0.76 vs. 0.69). However this was only a trend, not achieving significance amongst the three, the closest being volume vs. diameter (p > 0.15). This is similar to what Shah *et al *and Galanis *et al *have reported as correlations between uni and multi-dimensional radiological data in classifying progressive disease [19,20].

Furthermore, we show that a qualitative interpretation of any radiological progression one-month post therapy is associated with poor outcomes. However, this assessment is not acted upon clinically because of the confounding potential for treatment effect (or pseudoprogression), and our current inability (clinically and radiologically) to distinguish the two groups *apriori*. Many recent investigations have looked at the incidence and outcomes related to pseudoprogression [21-24]. Two Canadian studies by Roldan et al and Sanghera et al found rates of pseudoprogression of 40% and 32% respectively, and median survivals of 9.1 months and 31.2 months [22,23]. Another recent study by Gerstner et al found the pseudoprogression rate to be 57% with a median survival of 24.4 months, however their definition of pseuodprogression was at 3 months post-chemoRT [24], compared to 6 months in this study (and the two referenced previously). All three showed no significant difference in OS between those with pseudoprogression and those without ERP. The results from this study were in keeping with other literature, including a rate of pseudoprogression of 38.5% and a median survival of 19.0 months. There was also no survival benefit between pseudoprogressors and those patients with no ERP, however pseudoprgressors showed improved OS compared with true early progressors (median survival 19.0 mo vs. 7.2 mo, p < 0.01), in keeping with the results of Roldan and Sanghera [22,23]. This demonstrates that there is sufficient qualitative information in early structural imaging to help guide clinicians in identifying progressive vs. responsive disease, with the exception of pseudoprogression, a topic which is now finding its way into the realm of imaging response criteria.

Historically, quantitative imaging criteria was first addressed in 1979 by the WHO in their published guidelines [2]. Since then, RECIST v1.0 [13] was published in 2000 with subsequent revised criteria (version 1.1) in 2009 [14]. Each was developed in an attempt to standardize reporting and facilitate comparison of imaging response assessment within the context of clinical oncology trials [4,11], however the results of this study show that the ability to assess progressive disease via quantitative radiological data remains limited. We found that each of the MacDonald, RECIST and additional thresholds, both uni and multi-dimensional, while specific for progressive disease were highly insensitive. This translated into a poor correlation with both PFS at one year and OS at two years (Figure 4), therefore limiting their usefulness as endpoint surrogates in clinical trials. One obvious contributor to this effect is the issue of pseudoprogression, in that pseudoprogressors will always negatively impact the accuracy of progressive thresholds based on standard structural imaging. Recent updates in response assessment criteria by the RANO group (Response Assessment in Neuro-Oncology) have included an effort to address these challenges by developing guidelines specific to the management of brain tumors including parameters for disease progression [15]. They suggest deferring the determination of progressive disease until ≥ 12 weeks after the completion of RT, except in the case of a new lesion outside of the radiation field and/or pathology proven progressive disease within the original tumor site. This recommendation aims to defer a change in clinical management until pseuodprogression can be more reliably ruled out. However, as was mentioned previously the OS between pseudoprogressors identified at one month after RT is not significantly different from non-progressors, and therefore if these patients could be identified more readily, the truly progressive patients would avoid an additional 8 weeks of ineffective chemotherapy.

In contrast, metrics for defining *responsive *disease performed much better in terms of both PFS and OS (Figure 4), likely in part because identifying responders is not marred by the issue of pseudoprogression and also because intuitively, those with large reductions in tumor burden will do better than those without. Clinical trials showing evidence of radiological response in GBM are therefore likely to have an increased clinical relevance in terms of survival endpoints, than those focusing on progressive characteristics. This is contrary to the findings of Galanis *et al *who found that progressive disease to be more predictive of OS. This difference is probably multi-factorial, for one a variety of gliomas were included as compared to solely GBM as in this study. Secondly, the there was a smaller portion of responders in the Galanis study, likely owing in part to the addition of temozolomide to the treatment regiment in this study. Finally, the timing of the imaging was later in the Galanis study, 4 months post-induction of therapy as compared to one month post-RT in our study. This difference in timing may decrease the incidence of pseudoprogressors as a fraction may have already declared themselves as true early progressors by that point, thereby alleviating their negative statistical impact on the progressive imaging thresholds. If true, it is conceivable that optimizing the timing of post-therapy follow-up imaging could aid in of identification of pseudoprogressors. Our study only looked at a single imaging time point, however further investigation into multiple imaging time points would certainly be insightful. It is unlikely however that the answer to this challenging issue lies in timing along, and as such an array of research continues to look for potentially more robust and quantifiable solutions. Many groups have looked at the use of functional imaging modalities to augment standard anatomical information. The addition of perfusion and diffusion-weighted techniques are thought to be able to provide information about tumor activity as a potential biomarker of tumor progression [25]. As such, the role of functional MRI (diffusion-weighted and perfusion) is the subject of intense clinical investigation [26-33], and recent findings have shown that diffusion-weighted imaging can predict for OS and time-to-progression in high grade glioma [29,30]. Furthermore, recent results by Tsien *et. al*. have shown promise in using dynamic susceptibility contrast magnetic resonace imaging (DSC-MRI) and parametric response maps measuring relative cerebral blood volume to identify pseudoprogression from true progression during therapy [34]. The role of FLT-PET and molecular imaging is also being actively investigated as a potential modality for imaging tumor progression [35,36].

A primary limitation of our study lies in a relatively small sample size of prospectively recruited Glioblastoma patients. Our work must be further validated in a larger cohort for meaningful interpretation and future clinical translation. Furthermore, as was mentioned above, our study only investigated a single imaging time point (one month post-RT), additional imaging would be useful determining if there is an optimal time point, and what that might be. Our study cohort had a significantly higher median survival (26.2 mo. 95% CI 13.7 - not reached) than expected from the literature (14.6 mo. 95% CI 13.2 - 16.8 [37]). Finally, baseline imaging in the study was performed post-operatively, where resolving post-surgical changes may have been a potential confounding factor in the assessment of response. Strengths of this cohort include a typical and balanced population demographic in age, gender and size. Extent of surgery was also balanced with ~50% undergoing gross total resection and the remainder having either partial total resection or biopsy alone. The extended length of follow-up (median 22 months) was also beneficial to this study.

## Conclusion

We sought to evaluate early radiologic response criteria relevant to clinical outcomes in patients with GBM treated with concurrent chemotherapy and radiotherapy, and found that a qualitative clinical impression of radiologic progression at one month after therapy was predictive of poor outcomes despite the confounding factor of treatment effect (pseudoprogression). Quantitatively, we found that response metrics were more indicative of outcome than progressive indices and that there was a trend of volumetric data outperforming diameter or area thresholds, however significance was not reached in this case. Further investigation will focus on adding additional imaging time points as well as adjunct functional imaging to better understand progression features that may have a stronger predictive value than structural geometric indices alone.

## Competing interests

The authors declare that they have no competing interests.

## Authors' contributions

Conception and design: AG, ESK and CM. Provision of study materials or patients: ESK, NL, WM, BM, EY and CM. Collection and assembly of data: AG, ESK, JH, GL and CM. Data analysis and interpretation: AG, ESK, GL. Manuscript writing: AG, ESK, JH, NL and CM. Final approval of manuscript: AG, ESK, JH, GL, NL, BA, WM, EY and CM.

## Supplementary Material

Additional file 1 **Table 1: Sensitivity and specificity metrics in predicting 2 year overall survival according to various volume thresholds, from baseline to one month after RT**. Click here for file

Additional file 2 **Table 2: Sensitivity and specificity metrics in predicting 2 year overall survival according to various area thresholds, from baseline to one month after RT**. Click here for file

Additional file 3 **Table 3: Sensitivity and specificity metrics in predicting 2 year overall survival according to various diameter thresholds, from baseline to one month after RT**. Click here for file

## References

[B1] Macdonald DR Cascino TL Schold SC Jr Cairncross JG Response criteria for phase II studies of supratentorial malignant glioma J Clin Oncol 1990 8 7 1277 80 2358840 10.1200/JCO.1990.8.7.1277

[B2] WHO handbook for reporting results of cancer treatment Geneva (Switzerland) 1979

[B3] Perry JR Cairncross JG Glioma therapies: how to tell which work? J Clin Oncol 2003 21 19 3547 9 10.1200/JCO.2003.05.885 12913102

[B4] Suzuki C Jacobsson H Hatschek T Torkzad MR Boden K Eriksson-Alm Y Radiologic measurements of tumor response to treatment: practical approaches and limitations Radiographics 2008 28 2 329 44 10.1148/rg.282075068 18349443

[B5] van den Bent MJ Vogelbaum MA Wen PY Macdonald DR Chang SM End point assessment in gliomas: novel treatments limit usefulness of classical Macdonald's Criteria J Clin Oncol 2009 27 18 2905 8 10.1200/JCO.2009.22.4998 19451418 PMC2702230

[B6] Ruben JD Dally M Bailey M Smith R McLean CA Fedele P Cerebral radiation necrosis: incidence, outcomes, and risk factors with emphasis on radiation parameters and chemotherapy Int J Radiat Oncol Biol Phys 2006 65 2 499 508 10.1016/j.ijrobp.2005.12.002 16517093

[B7] Brandes AA Franceschi E Tosoni A Blatt V Pession A Tallini G MGMT promoter methylation status can predict the incidence and outcome of pseudoprogression after concomitant radiochemotherapy in newly diagnosed glioblastoma patients J Clin Oncol 2008 26 13 2192 7 10.1200/JCO.2007.14.8163 18445844

[B8] Taal W Brandsma D de Bruin HG Bromberg JE Swaak-Kragten AT Smitt PA Incidence of early pseudo-progression in a cohort of malignant glioma patients treated with chemoirradiation with temozolomide Cancer 2008 113 2 405 10 10.1002/cncr.23562 18484594

[B9] Brandsma D Stalpers L Taal W Sminia P van den Bent MJ Clinical features, mechanisms, and management of pseudoprogression in malignant gliomas Lancet Oncol 2008 9 5 453 61 10.1016/S1470-2045(08)70125-6 18452856

[B10] Gonzalez J Kumar AJ Conrad CA Levin VA Effect of bevacizumab on radiation necrosis of the brain Int J Radiat Oncol Biol Phys 2007 67 2 323 6 10.1016/j.ijrobp.2006.10.010 17236958

[B11] Henson JW Ulmer S Harris GJ Brain tumor imaging in clinical trials AJNR Am J Neuroradiol 2008 29 3 419 24 10.3174/ajnr.A0963 18272557 PMC8118884

[B12] Lang FF Gilbert MR Puduvalli VK Weinberg J Levin VA Yung WK Toward better early-phase brain tumor clinical trials: a reappraisal of current methods and proposals for future strategies Neuro Oncol 2002 4 4 268 77 12356357 10.1093/neuonc/4.4.268 PMC1920662

[B13] Therasse P Arbuck SG Eisenhauer EA Wanders J Kaplan RS Rubinstein L New guidelines to evaluate the response to treatment in solid tumors. European Organization for Research and Treatment of Cancer, National Cancer Institute of the United States, National Cancer Institute of Canada J Natl Cancer Inst 2000 92 3 205 16 10.1093/jnci/92.3.205 10655437

[B14] Eisenhauer EA Therasse P Bogaerts J Schwartz LH Sargent D Ford R New response evaluation criteria in solid tumors: revised RECIST guideline (version 1.1) Eur J Cancer 2009 45 2 228 47 10.1016/j.ejca.2008.10.026 19097774

[B15] Wen PY Macdonald DR Reardon DA van den Bent MJ Chang SM Updated response assessment criteria for high-grade gliomas: response assessment in neuro-oncology working group J Clin Oncol 2010 28 11 1963 1972 10.1200/JCO.2009.26.3541 20231676

[B16] DeLong ER DeLong DM Clarke-Pearson DL Comparing the areas under two or more correlated receiver operating characteristic curves: a nonparametric approach Biometrics 1988 44 837 845 10.2307/2531595 3203132

[B17] Lamborn KR Yung WK Chang SM Wen PY Cloughesy TF DeAngelis LM Progression-free survival: an important end point in evaluating therapy for recurrent high-grade gliomas Neuro Oncol 2008 10 2 162 70 10.1215/15228517-2007-062 18356283 PMC2613818

[B18] Shankar LK Van den AA Yap J Benjamin R Scheutze S FitzGerald TJ Considerations for the use of imaging tools for phase II treatment trials in oncology Clin Cancer Res 2009 15 6 1891 7 10.1158/1078-0432.CCR-08-2030 19276276

[B19] Shah S Kesari S Xu R Batchelor T O'Neill A Hochberg F Levy B Bradshaw J Wen P Comparison of linear and volumetric criteria in assessing tumor response in adult high-grade gliomas Neuro Onc 2006 8 38 46 10.1215/S1522851705000529 PMC1871928 16443946

[B20] Galanis E Buckner JC Maurer MJ Sykora R Castillo R Ballman KV Erickson BJ Validation of neuroradiologic response assessment in gliomas: Measurement by RECIST, two-dimensional, computer-assisted tumor area, and computer-assisted tumor volume methods Neuoro Onc 2006 8 2 156 65 10.1215/15228517-2005-005 PMC1871930 16533757

[B21] Sorensen AG Batchelor TT Wen PY Zhang WT Jain RK Response criteria for glioma Nat Clin Prac Onc 2008 11 5 634 44 10.1038/ncponc1204 PMC4795821 18711427

[B22] Roldán GB Scot JN Hamilton MG Easaw JC Population-based study of pseudoprogression after chemotadiotherapy in GBM Can J Neurol Sci 2009 36 617 22 19831132 10.1017/s0317167100008131

[B23] Sanghera P Perry J Davey P Tsao MN Pseudoprogression following chemotadiotherapy for glioblastoma multiforme Can J Neurol Sci 2010 37 36 42 20169771 10.1017/s0317167100009628

[B24] Gerstner ER McNamara MB Norden AD LaFrankie D Wen PY Effect of adding temozolomide to radiation therapy on the incidence of pseudo-progression J Neuroncol 2009 94 97 101 10.1007/s11060-009-9809-4 19221865

[B25] Provenzale JM Mukundan S Barboriak DP Diffusion-weighted and perfusion MR imaging for brain tumor characterization and assessment of treatment response Radiology 2006 239 3 632 49 10.1148/radiol.2393042031 16714455

[B26] Cao Y Tsien CI Nagesh V Junck L Ten HR Ross BD Survival prediction in high-grade gliomas by MRI perfusion before and during early stage of RT [corrected] Int J Radiat Oncol Biol Phys 2006 64 3 876 85 10.1016/j.ijrobp.2005.09.001 16298499

[B27] Chang S Clarke J Wen PY Novel Imaging Response Assessment for Drug Therapies in Recurrent Malignant Glioma J Clin Onc 2009 107 11

[B28] Park I Tamai G Lee MC Chuang CF Chang SM Berger MS Patterns of recurrence analysis in newly diagnosed glioblastoma multiforme after three-dimensional conformal radiation therapy with respect to pre-radiation therapy magnetic resonance spectroscopic findings Int J Radiat Oncol Biol Phys 2007 69 2 381 9 10.1016/j.ijrobp.2007.03.019 17513061 PMC2377157

[B29] Hamstra A Chenevert T Moffat B Evaluation of the functional diffusion map as an early biomarker of time-to-progression and overall survival in high-grade glioma Proc Nat Acad Scien 2005 102 46 16759 64 10.1073/pnas.0508347102 PMC1276616 16267128

[B30] Hamstra DA Galbán CJ Chenevert TL Functional diffusion map as an early imaging biomarker for high-grade glioma: correlation with conventional radiologic response and overall survival J Clin Oncol 2008 26 3387 94 10.1200/JCO.2007.15.2363 18541899 PMC3266717

[B31] Chenevert TL Stegman LD Taylor JM Diffusion magnetic resonance imaging: An early surrogate marker of therapeutic efficacy in brain tumors J Natl Cancer Inst 2000 92 2029 36 10.1093/jnci/92.24.2029 11121466

[B32] Provenzale JM York G Serajuddin H Correlation of relative permeability and relative cerebral blood volume in high-grade cerebral neoplasms Am J Roentgenol 2006 187 1036 42 10.2214/AJR.04.0676 16985154

[B33] Bian W Khayal IS Nelson SJ Multiparametric characterization of grade 2 glioma subtypes using magnetic resonance spectroscopic, perfusion and diffusion imaging Transl Oncol 2009 2 271 80 19956389 10.1593/tlo.09178 PMC2781083

[B34] Tsien C Galbán C Chenevert T Parametric Response Map As an Imaging Biomarker to Distinguish Progression From Pseudoprogression in High-Grade Glioma J Clin Oncol 2010 28 2293 2299 10.1200/JCO.2009.25.3971 20368564 PMC2860441

[B35] Larson SM Schwartz LH 18F-FDG PET as a candidate for "qualified biomarker": functional assessment of treatment response in oncology J Nucl Med 2006 47 6 901 3 16741296

[B36] Backes H Ullrich R Jacobs AH Noninvasive quantification of (18)F-FLT human brain PET for the assessment of tumour proliferation in patients with high-grade glioma Eur J Nucl Med Mol Imaging 2009 26 1960 67 10.1007/s00259-009-1244-4 PMC2779371 19672593

[B37] Stupp R Mason WP van den Bent MJ Radiotherapy plus concomitant and adjuvant temozolomide for glioblastoma N Engl J Med 2005 352 987 996 10.1056/NEJMoa043330 15758009

